# Implementing a Registry Federation for Materials Science Data Discovery

**DOI:** 10.5334/dsj-2021-015

**Published:** 2021

**Authors:** RAYMOND L. PLANTE, CHANDLER A. BECKER, ANDREA MEDINA-SMITH, KEVIN BRADY, ALDEN DIMA, BENJAMIN LONG, LAURA M. BARTOLO, JAMES A. WARREN, ROBERT J. HANISCH

**Affiliations:** National Institute of Standards and Technology, Material Measurement Laboratory, Office of Data and Informatics, Gaithersburg, MD, United States of America; National Institute of Standards and Technology, Material Measurement Laboratory, Office of Data and Informatics, Gaithersburg, MD, United States of America; National Institute of Standards and Technology, Information Services Office, Gaithersburg, MD, United States of America; National Institute of Standards and Technology, Information Technology Laboratory, Gaithersburg, MD, United States of America; National Institute of Standards and Technology, Information Technology Laboratory, Gaithersburg, MD, United States of America; National Institute of Standards and Technology, Information Technology Laboratory, Gaithersburg, MD, United States of America; Northwestern University, Center for Hierarchical Materials Design, Evanston, IL, United States of America; National Institute of Standards and Technology, Material Measurement Laboratory, Gaithersburg, MD, United States of America; National Institute of Standards and Technology, Material Measurement Laboratory, Office of Data and Informatics, Gaithersburg, MD, United States of America

**Keywords:** registry, informatics, data discovery, metadata, OAI-PMH

## Abstract

As a result of a number of national initiatives, we are seeing rapid growth in the data important to materials science that are available over the web. Consequently, it is becoming increasingly difficult for researchers to learn what data are available and how to access them. To address this problem, the Research Data Alliance (RDA) Working Group for International Materials Science Registries (IMRR) was established to bring together materials science and information technology experts to develop an international federation of registries that can be used for global discovery of data resources for materials science. A resource registry collects high-level metadata descriptions of resources such as data repositories, archives, websites, and services that are useful for data-driven research. By making the collection searchable, it aids scientists in industry, universities, and government laboratories to discover data relevant to their research and work interests.

We present the results of our successful piloting of a registry federation for materials science data discovery. In particular, we out a blueprint for creating such a federation that is capable of amassing a global view of all available materials science data, and we enumerate the requirements for the standards that make the registries interoperable within the federation. These standards include a protocol for exchanging resource descriptions and a standard metadata schema for encoding those descriptions. We summarize how we leveraged an existing standard (OAI-PMH) for metadata exchange. Finally, we review the registry software developed to realize the federation and describe the user experience.

## INTRODUCTION: TARGETED PROBLEM SPACE AND GOALS

The Materials Science and Engineering (MSE) research domain is exceptionally broad and interdisciplinary with its origins most directly from metallurgy, ceramics, and polymer science, but also with important ties to other disciplines such as physics, chemistry, chemical engineering, geology, electronics, optics, and biology. As a global community, MSE is expanding rapidly worldwide through the establishment of large, multi-institutional academic research centers, government laboratories, industrial consortia, and computing facilities. MSE researchers often need to answer complex questions such as “What structural properties and processing methods are required to develop new lightweight materials for vehicles that significantly improve fuel efficiency yet meet safety standards satisfied by traditional materials in use today?” To this end we have seen the creation of programs such as the Materials Genome Initiative (MGI, https://www.nist.gov/mgi) in the US and comparable international materials-focused initiatives in China, Europe, and Japan. These initiatives share a consistent goal: decrease the cost and time to develop new materials by a factor of two through more effective discovery, access, and interoperability of experimental and simulation data.

It is under the drive of these initiatives that we have seen increased efforts to make materials science data accessible via the web. Newer data projects like the Materials Project (Jain et al. 2013) and Materials Commons (Puchala et al. 2016) from academia and industrial initiatives join a legacy of existing data resources that in some cases pre-date the web. In this rapidly evolving climate for data, materials scientists and engineers who might want to make use of the growing digital wealth of information have lacked a comprehensive mechanism for learning what data even exist. At best, we had manually curated web pages that simply listed the most well-known projects (in the eyes of the curator). Such a web site is not likely to achieve comprehensive coverage of the available data in the face of a growing data activity, particularly for the “long-tail” of published data.

In response to the challenge of finding data, we worked within the context of the Research Data Alliance (RDA, https://www.rd-alliance.org/) and its RDA/CODATA Materials Data, Infrastructure, and Interoperability Interest Group.^1^ In collaboration with materials science researchers and data management specialists, we created the International Materials Resource Registries (IMRR) Working Group. The IMRR WG assembled MSE domain experts representing different regions and sectors, including Asia, Europe, and North America. As part of establishing this federation, we must identify and define the standards and profiles necessary to operate in an open and scalable way. This paper reports on the output of that working group.

This working group’s largest accomplishment and its approach to data discovery was the development of a *resource registry* for materials science. We define a registry to be a searchable collection of data resource descriptions, similar to a library’s catalog. A *data resource* is typically a data collection of some kind, like a database, a data publication, or a data repository; however, in general, it can refer to anything that is useful for doing data-enabled science, including software, services, web portals, informational web sites, and even the organizations that provide tools and data. Data resource descriptions come in the form of digital metadata records that include, most importantly, a URL for getting access and more information about the resource. By making these descriptions searchable, researchers have a way to discover resources related to a particular scientific topic.

For the scope of this project, we have primarily restricted ourselves to what can be referred to as high-level resources like repositories, databases, and web portals. We have not focused on individual datasets, data records, or measurements. The aim here is to direct users to the home pages for data that are supported by the people and organizations who have made the data available; there, the users can leverage the collection-specific tools to delve deeper to find the individual datasets or records that they need (rather than circumventing those tools). Another reason to keep our registry at a high-level view of what data are available is that additional challenges arise if the registry must scale up to supporting potentially millions of records.

One of those challenges is keeping the registry continuously up to date. Active data resources could be continuously adding new data, making it more likely that the registry goes out of date; on the other hand, the metadata describing web sites and large data aggregations will change more slowly. Most importantly, we feel that the metadata that distinguishes fine-grain resources like individual datasets or records will be much more diverse, more challenging to integrate, and is best curated by the providers of that data in the systems they built to handle the associated metadata.

We note that while our registry contains only coarse-grained view of the materials science data that is out there, this does not prevent us from using it to discover and download individual datasets. As an example, from the astronomical community, the virtual observatory framework features a registry as the first step in an automated data discovery process: the registry is used to find the repositories and portals where data is served (Dower, 2018). Users can be directed to those portals; however, if the sites have also registered search services, those sites can be queried automatically to find individual datasets or measurements. Since the registry itself features its own application programming interface (API) for searching, third-party tools can carry out the searches on behalf of users without them realizing that a registry is involved. With a layered approach to discovery like this, building a community registry represents a practical, tractable first step that is still useful on its own.

This paper focuses on the registry framework that was adopted for the Working Group’s working demonstration of a registry federation for discovering data resources for MSE research. It is based on the architecture and experience built by the International Virtual Observatory Alliance (IVOA; Dower, 2018) and attempts to generalize their approach for application to any discipline. In particular, extract the key requirements that makes a registry federation work. In a complementary paper (Medina-Smith, 2021), we describe the metadata schema that was used to describe the MSE data resources.

## THE FEDERATED REGISTRY FRAMEWORK

Our model for a registry-mediated data search process is based on a *federated* architecture (adapted from the model used by the Virtual Observatory in the astronomy domain). Specifically, this means that there is no one master registry; rather, we have a network of registries working together. Any registry can pull in the resource descriptions from each of the other registries using a common metadata exchange protocol in order to build a comprehensive collection of resource descriptions of all known resources in the network. It may then make this collection searchable.

From a technical perspective, the federation is an open one: any organization can host a registry as a means of advertising their own resources to the world. We refer to a registry whose primary function is to export resource descriptions out to the federation as *publishing registry.* In this role, the registry providers take responsibility for creating and curating the descriptive records for a specific subset of resources. By “curating”, we mean keeping the records accurate in their content, up-to-date, and compliant with the metadata standards in use. If the registry is operated by a data center that provides a variety of resources—databases, data collections, services, and perhaps a portal to navigate them all—the registry would then curate the records describing the resources it provides. A registry might also be run by a particular sub-community in a domain, curating records on the behalf of its constituents; in our pilot, the Center for Hierarchical Materials Design (CHiMaD, https://chimad.northwestern.edu/) registry manages records for resources provided by the Center’s member organizations. When a data provider only shares a few resources (say, a single database or a few datasets), it may not make sense for them to also operate a registry; instead, there can be publishing registries that host records on community providers’ behalf. In our pilot, the NIST registry plays that role: the registry web portal allows a provider to login, create resource descriptions, publish them out, and update them over time.

The other important role of a registry, of course, is making the resource descriptions searchable. Not every registry in the federation needs to do this, so we distinguish this role by referring to *searchable registries*. In our model, registries are *not* obligated to provide search capabilities in the same way, particularly through their user-oriented web sites; rather, a searchable registry can tailor its search services to the primary audience they serve. (There is great value in providing a common search API for powering remote clients; however, this was beyond the scope of the goals of the working group.)

## WHY FEDERATE?

We recommend a federated model as part of an approach to sustainable infrastructure. In particular, a federation brings these key features:

1. Distributed metadata curation
  With a single, centralized registry, there is a danger that registry records become effectively divorced from the people that care about the things being described, and so it is common under such a model for records to be inaccurate and become out of date. In the federated model, the curation of the registry records can be distributed across the community and kept closer to the experts responsible for providing the resources the records describe. While record curation will still be a sociological challenge, it is made more tractable when more of the community can be involved.
2. No single point of failure
  When there are multiple registries that have complete collections of resource descriptions, discovery services need not completely shut-down when one registry goes off-line: users and search clients have alternate registries they can connect to. Robustness to registry failure can also address sustainability concerns when the federation spans the globe.
3. Allows for innovation
  The registry federation need not present a one-size-fits-all solution for data discovery. That is, searchable registries can specialize their capabilities to a particular sub-community who it is their mission to serve, whether it is in the search interface that is presented to the user or the way the records are indexed.

### REQUIREMENTS FOR INTEROPERABLE REGISTRIES

To interoperate, federated registries must have *(1)* a common metadata exchange protocol and *(2)* a common metadata schema and format for passing records within that protocol. Multiple open standards exist today which can be adopted to define a registry federation; because the standards are general and not community- or application-specific, additional requirements are needed to define the profile on those standards for a particular community:

1. The profile on the common metadata exchange protocol should,
  - Provide a means for identifying the record format(s) and schema(s) that can be used to encode resource descriptions. (Often the metadata schema and format are coupled together as a single standard.)
  - Set a distinction between the records that have been created and curated by the registry sharing their records, and the records that it has harvested from other registries; the protocol should allow (or require) delivery of only the former. This ensures that harvesters only receive one copy of a record from its definitive source.
  - Be able to communicate that a resource (and its resource description) is no longer available.
  - Require minimal validation of records before they are made available to users and clients (for searching, harvesting, etc.). In a distributed system, when something goes wrong, it is often unclear to users who is responsible. By requiring validation of resource descriptions before exporting them from their registry of origin, problems in the resource records can be detected close to where they are best fixed.
2. The common metadata format should:
  - Be openly defined,
  - Have a unique identifier associated with it, and
  - Be validatable.

When a community standardizes the requirements for participating in a registry federation, these are the minimum features the community must define. We note that other best practices regarding the definition and use of a metadata standard should be applied, including connecting the metadata schema to community-recognized vocabulary and semantics; these could also be part of the standard. See Medina-Smith, et al. (Medina-Smith, 2021) for further discussion.

We note that the need for a common metadata schema is specifically for unifying the high-level discovery process. Within our framework, we want to enable the creation of rich and varied applications for indexing and searching through the resource records; thus, developers need to know how to extract particular kinds of information across all records. Consequently, a single, common schema makes this possible in the simplest way. Nevertheless, throughout the community that produces this metadata, many schemas and formats are in use. Some schemas are adopted locally because they capture information important to a particular sub-community (say, geographic locations or astronomical positions). It is not the intention of this framework to cut off access to this richer information; rather, this richer information can be more easily leveraged if the metadata exchange protocol is capable of sharing records in multiple formats.

## OAI-PMH AS A STANDARD EXCHANGE PROTOCOL

For our pilot for the Materials Science community, we chose the Open Archives Initiative Protocol for Metadata Harvesting (OAI-PMH) (Lagoze et al. 2002). This was chosen for two main reasons. First, as an XML-based protocol, it is well suited for transmitting our XML-formatted metadata records. Second, it is broadly used across many communities and has an established track record for successfully enabling interoperability between data centers, registries, and end-user tools. Finally, the protocol not only meets the requirements set out in the previous section, but provides additional features that make it an efficient means to exchange metadata, including incremental harvesting (i.e. harvesting new or changed records since some given date), record paging, and support for multiple metadata schemas.

OAI-PMH is a “pull” protocol: a registry (or other consumer) wanting records—the *harvester*—asks for new or changed records from another registry and pulls them in to its own collection. In this protocol model, the harvester gets to choose which other registries it will collect records from and when. This is in contrast to a “push” protocol, where the registry sends its records out to other interested registries; this requires that the registry know in advance who wants its records (its *subscribers*).

OAI-PMH is quite flexible in how it can be used; however, additional care must be taken to ensure that metadata records get replicated in a timely and efficient manner. We recommend a profile like that defined and used by the registry standard of the International Virtual Observatory Alliance (IVOA) (Dower 2018) which has the following features:

- a standard name for the common metadata schema/format (“metadata prefix” in OAI-PMH terminology) that harvesters use to request records in the federation’s common format; thus, other formats can be named and requested independently.
- a standard name for a subset of the available records (a “set”, in OAI-PMH terminology) indicating records that originate from the harvestee. A registry, in general, will contain records it created and curates, as well as records it harvested from other registries. To avoid harvesting duplicate records from different registries, the harvest requests the subset that contains only the former using the standard set name.

Another key feature of the IVOA framework is a “registry of registries”. This service’s job is to list the harvestable registries that are in the federation. Thus, to get a complete collection of all records in the federation, a harvester first discovers the registries and the requests from each their unique set of records that they curate. As we describe in the next section, our nascent federation currently only has two registries in it, so registry discovery is not so critical in this phase of our pilot.

## THE REGISTRY APPLICATION

With the Information Systems Group at the NIST Information Technology Laboratory, we developed a registry application based on their existing software, the NIST Materials Data Curation System (Dima et al. 2016). The registry software (which is still under active development) is available from the GitHub repository, usnistgov/nmrr (Brady et al. 2019). The software runs as a web application, and in our pilot, there are two instances in operation:

- http://materials.registry.nist.gov/
- http://mrr.materialsdatafacility.org/

The WG members at NIST have populated its registry with records describing resources created and maintained at NIST. They have also opened up the registry for describing resources external to NIST. Many of these latter records were created by the NIST curators on behalf of external organizations as a means to seed the collection and attract interest; however, a number of external users have created accounts with the registry to create their own descriptions of resources they provide (described below). The second registry operates similarly: it automatically generates records about data holdings in the Materials Data Facility as well as providing registration access to its researchers in the CHiMaD collaboration. Both registries are configured to daily pull in new and updated records from the other registry.

Any researcher looking for data resources (e.g. repositories of DFT calculations) can visit the website, access the search page, submit simple keyword-based search queries, and receive back a listing of matching resources. The search results page provides faceted browsing of the results that leverages the controlled vocabulary. The user can drill down into subsets of results by clicking on different resource attributes and their categories. For example, the user can select particular resource types (like databases or software) that provide experimental data related to a topic of interest. Each search result includes a link to the resource’s native landing page as provided by the data provider; thus, the user can now visit the resource’s web site directly, download data or use the native tools provided by the data provider.

Data providers can also visit the website to register the existence of their resources. When they create an account, they have a space where they can create descriptions of different kinds of resources. After selecting a resource type, they are presented with a form where they can enter the metadata. In particular, the materials science metadata is presented as checkboxes with expandable detail.

The application exploits the general capabilities of the underlying curation software. In particular, both the resource registration form (where resource descriptions are created) and the search results page (where hits can be filtered) are generated on the fly based directly from the XML Schema document. This means that the application can be easily adapted to other schemas and domains. In fact, NIST has re-used this software and its underlying model to set up registry federations for other domain communities, including metrology (http://imrr.bipm.org/) and green-house gas research (https://ghgr.nist.gov/).

The registry application also features a rich API for both searching its contents and uploading new records. We envision the latter being important for integrating a registry with a data center’s infrastructure: the API can be used to automatically push descriptions of resources based on metadata from the center’s native systems. Because the registry application also supports an OAI-PMH harvesting services it can expose this service to other registries and the application can be configured to harvest from other OAI-PMH services at regular intervals.

As of this writing, the federated registry contains more than 350 records describing resources representing records from 50+ organizations.^2^ Many of the resources describe software; this reflects a collaboration with the Materials Genome Initiative (MGI) in which we included records from the MGI Code Catalog. The registry also currently describes a number of databases, repositories, project archives, services, and portals and web sites. Further work is focused on expanding the contents of the registry system, particularly organizations and services.

## FUTURE PLANS

Although this working group has finished its activities, we hope that the community we have built around this work can continue to grow. Going forward, NIST plans to lead an effort to involve more providers in registering their resources as well as recruit centers that can host their own registries and integrate them into the registry federation for materials science.

With this expectation, there are a number of improvements and new pilots that we envision for improving registry-based data discovery. As mentioned above, NIST continues to develop the registry software. The software’s core (now called the Configurable Data Curation System) is being updated to make it more modular and, therefore, more adaptable to new domains and applications (Brady 2018). As part of this transformation, NIST is moving more to an open source development process to leverage contributions from the growing external user community. In this new phase of development, we hope to address a number of critical and desired features of the software. In particular, we would like to:

- Provide improved XML Schema support that can enable community-based standardization of schemas.
- Improve support for various persistent identifiers and facilitate robust linking of related resources (such as linking services to the organizations that provide them).
- Add support for evolving schemas with robust validation.
- Explore support for other metadata format types such as JSON.

We would also like to explore new pilots in advanced data discovery. An important one would be connecting the registry (which describes only large or high-level resources) with more fine-grained discovery services provided by data centers. Data providers can already register web services they provide. We would like to enrich their descriptions so that the registry can recognize certain kinds of search services; in such a case, the registry—or any third-party tool using the registry—could automatically call the services on behalf of the user. In this way, a user might submit a query to the registry, and the registry can pass on that query to those services likely to have data. This tiered model for data discovery is used by the Virtual Observatory to drill down to individual datasets that can be downloaded or individual database records.

## Figures and Tables

**Figure 1:**
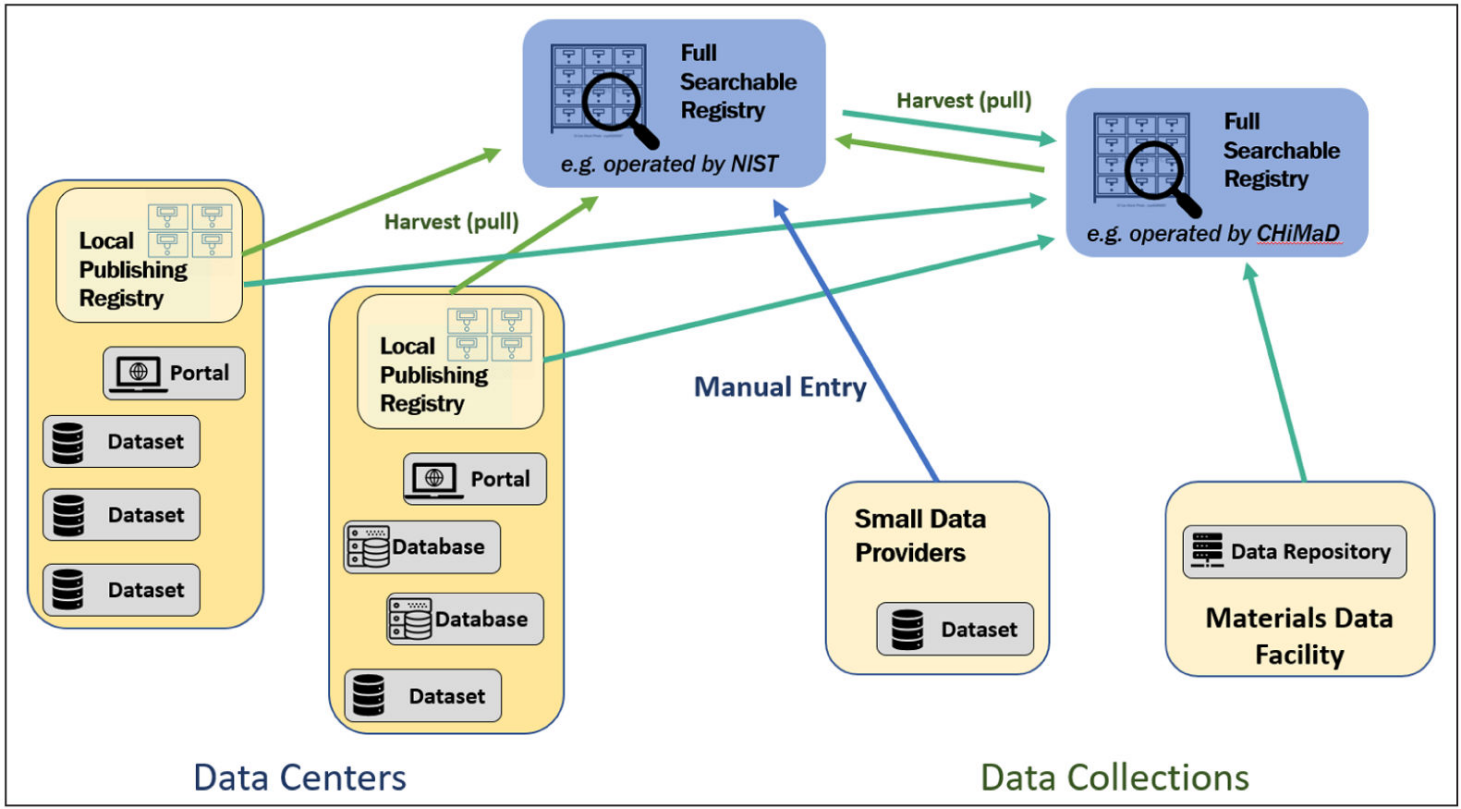
Record interchange within the registry federation. Searchable registries harvest resource descriptions from all publishing registries via a standard protocol. Some registries, like the one operated by CHiMaD, can manage records for a particular sub-community, possibly pulling in records through customized APIs. Other registries, like the one operated at NIST, can serve the at-large community where providers can create and maintain records through a GUI interface.

**Figure 2:**
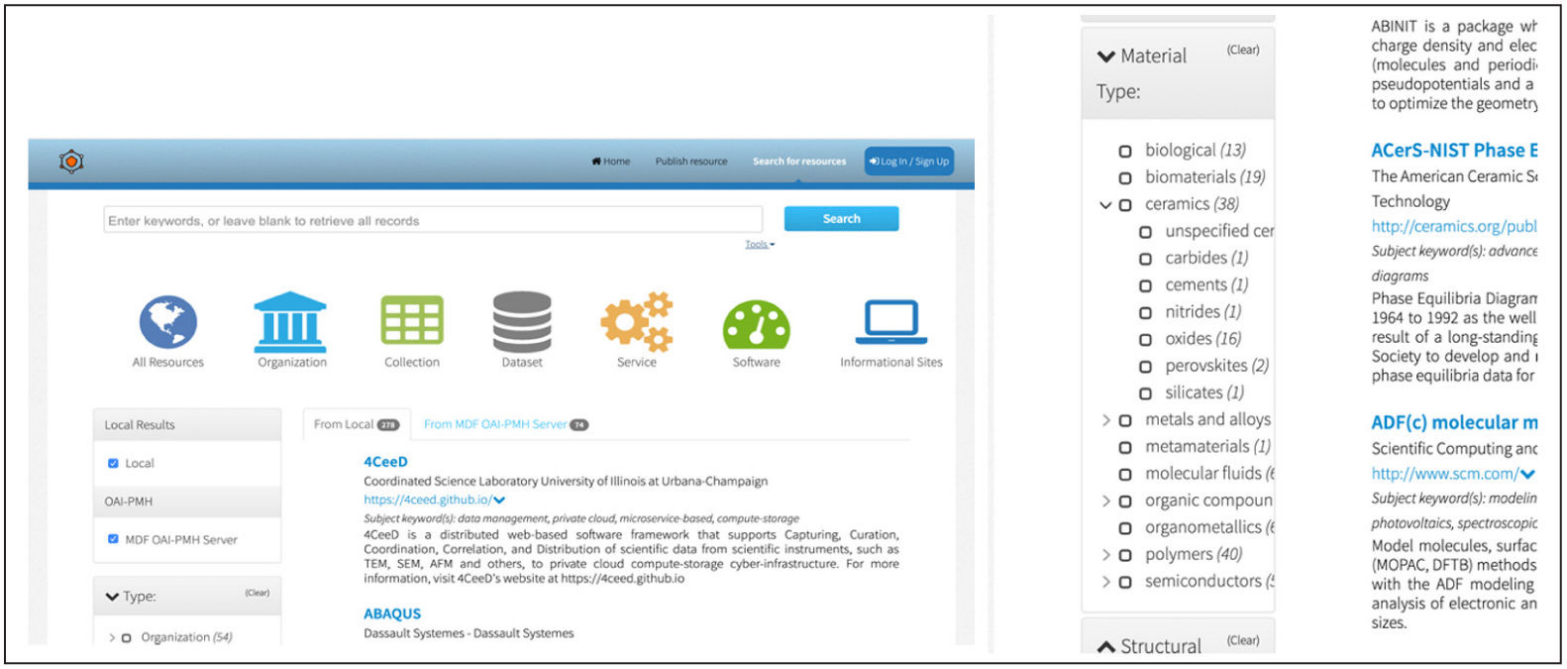
Registry search results. (left) Example of the search results page with faceted browsing filters to the left. (right) A zoomed view of the Material Type filters showing how general type, “ceramics”, can be clarified with more specific types of ceramics.

**Figure 3:**
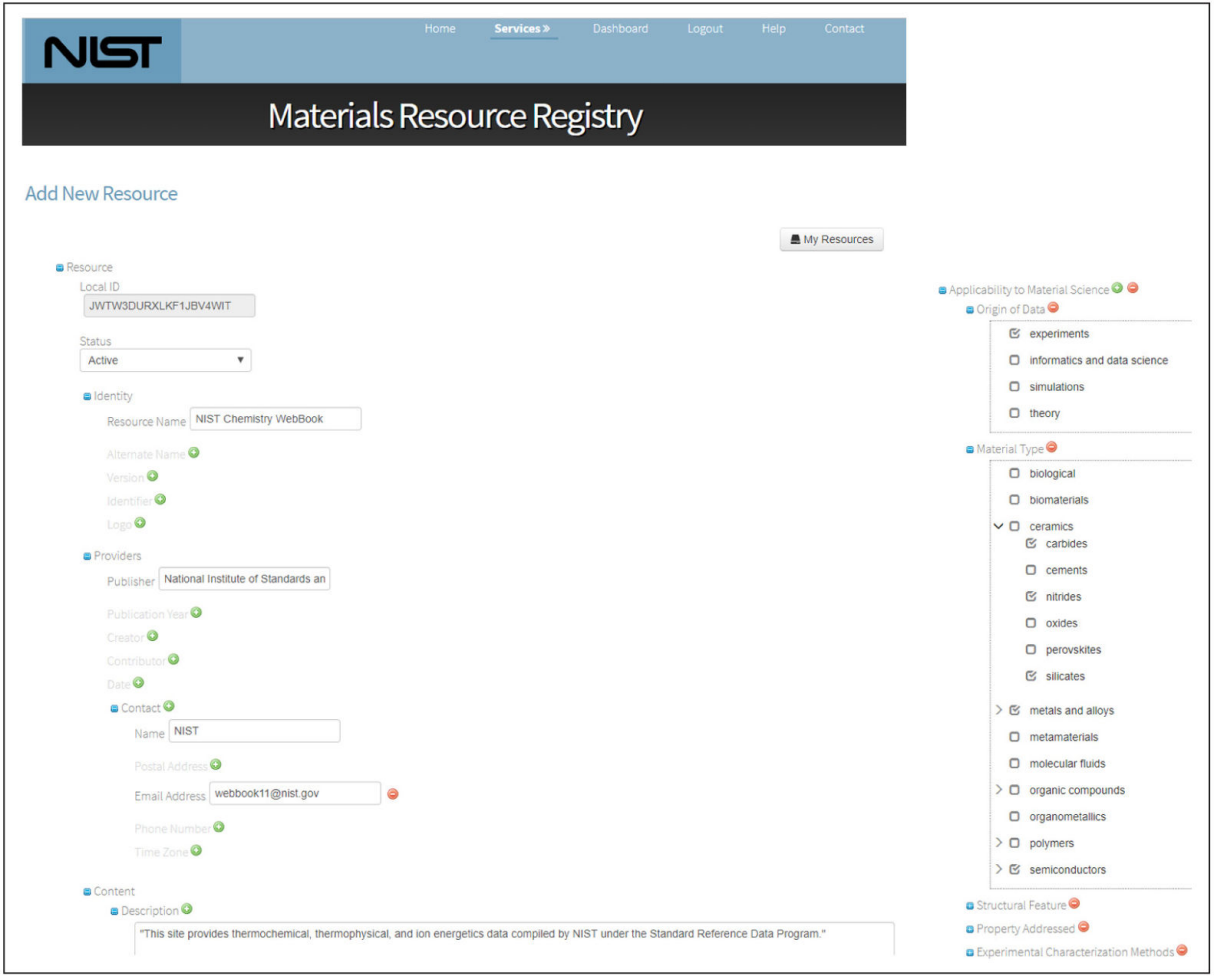
Resource Registration Form. (left) A portion of the form used to create a resource description and which is automatically generated from the schema. (right) Detail of the portion of the form where Material Science attributes can be selected; these terms come from the Materials Vocabulary.

## Footnotes

1 RDA/CODATA Material Data Infrastructure, and Interoperability Interest Group: https://www.rd-alliance.org/groups/rdacodata-materials-data-infrastructure-interoperability-ig.html.

2 We note that many of these were registered by NIST WG members on behalf of those organizations to help seed the registry and attract their interest.
